# Association of *KCNJ11* and *ABCC8* single-nucleotide polymorphisms with type 2 diabetes mellitus in a Kinh Vietnamese population

**DOI:** 10.1097/MD.0000000000031653

**Published:** 2022-11-18

**Authors:** Nam Quang Tran, Steven D. Truong, Phat Tung Ma, Chi Khanh Hoang, Bao Hoang Le, Thang Tat Ngo Dinh, Luong Van Tran, Thang Viet Tran, Linh Hoang Gia Le, Khuong Thai Le, Hien Thanh Nguyen, Hoang Anh Vu, Thao Phuong Mai, Minh Duc Do

**Affiliations:** a Department of Endocrinology, Faculty of Medicine, University of Medicine and Pharmacy at Ho Chi Minh City, Vietnam; b Department of Endocrinology, University Medical Center, University of Medicine and Pharmacy at Ho Chi Minh City, Vietnam; c Department of Medicine, School of Medicine, Stanford University, USA; d Center for Molecular Biomedicine, University of Medicine and Pharmacy at Ho Chi Minh City, Vietnam; e Department of Medical Laboratory Technology, University of Medicine and Pharmacy at Ho Chi Minh City, Vietnam; f Department of Physiology-Pathophysiology-Immunology, Faculty of Medicine, University of Medicine and Pharmacy at Ho Chi Minh City, Vietnam.

**Keywords:** *ABCC8*, Genetic association, *KCNJ11*, Kinh Vietnamese, type 2 diabetes mellitus

## Abstract

Type 2 diabetes mellitus (T2DM) is a genetically influenced disease, but few studies have been performed to investigate the genetic basis of T2DM in Vietnamese subjects. Thus, the potential associations of *KCNJ11* and *ABCC8* single nucleotide polymorphisms (SNPs) with T2DM were investigated in a Kinh Vietnamese population. A cross-sectional study consisting of 404 subjects including 202 T2DM cases and 202 non-T2DM controls was designed to examine the potential associations of 4 *KCNJ11* and *ABCC8* SNPs (rs5219, rs2285676, rs1799859, and rs757110) with T2DM. Genotypes were identified based on restriction fragment length polymorphism and tetra-primer amplification refractory mutation system polymerase chain reaction. After statistically adjusting for age, sex, and BMI, rs5219 was found to be associated with an increased risk of T2DM under 2 inheritance models: codominant (OR = 2.15, 95% confidence intervals [CI] = 1.09–4.22) and recessive (OR = 2.08, 95%CI = 1.09–3.94). On the other hand, rs2285676, rs1799859, and rs757110 were not associated with an increased risk of T2DM. Haplotype analysis elucidated a strong linkage disequilibrium between the 3 SNPs, rs5219, rs2285676, and rs757110. The haplotype rs5219(A)/rs2285676(T)/rs757110(G) was associated with an increased risk of T2DM (OR = 1.42, 95%CI = 1.01–1.99). The results show that rs5219 is a lead candidate SNP associated with an increased risk of developing T2DM in the Kinh Vietnamese population. Further functional characterization is needed to uncover the mechanism underlying the potential genotype-phenotype associations.

## 1. Introduction

According to the International Diabetes Federation, in 2021, diabetes affected approximately 10.5% of the world’s population-or 536.6 million people.^[1]^ Of this considerable number, more than 90% had type 2 diabetes mellitus (T2DM), a metabolic disorder characterized by insulin deficiency, insulin resistance, or both.^[2]^ Given T2DM’s increasing prevalence, studying the mechanisms leading to T2DM’s pathogenesis is imperative.

Previous studies have shown that East Asian and South Asian populations are genetically predisposed to T2DM.^[3–5]^ Compared to Caucasian individuals, East Asians and South Asians are more likely to develop T2DM at a lower body mass index (BMI).^[6,7]^ However, genetic data on Southeast Asian populations are severely lacking, especially within the context of T2DM. Given the complex pathophysiology of T2DM, as expected, numerous candidate genetic markers have been shown to be associated with this disease. Nevertheless, the results of these genetic studies are inconsistent, which can potentially be explained by inter-ethnic differences in the pathophysiology of T2DM. Recently, a lead candidate single nucleotide polymorphism (SNP), rs266729 in *ADIPOQ*, was shown to be associated with T2DM and metabolic syndrome in Kinh Vietnamese.^[8]^ These results show that the adiponectin pathway plays a role in the mechanism of T2DM. As insulin deficiency is a major mechanism in T2DM reported in Asian populations, we chose to study candidate SNPs in *KCNJ11* and *ABCC8* that may predispose the Kinh Vietnamese population to T2DM.

The *KCNJ11* and *ABCC8* genes encode the inwardly rectifying potassium ion channel (Kir6.2) and sulfonylurea receptor 1 (SUR1), respectively.^[9]^ These 2 subunits form the ATP-sensitive K^ + ^protein, an ion channel that plays a pivotal role in metabolism, facilitating insulin production and secretion.^[10]^ Polymorphisms of *KCNJ11* and *ABCC8* have been shown to be associated with T2DM.^[11,12]^ Among SNPs in *KCNJ11*, rs5219, and rs2285676 were chosen for this study as they have been shown to be consistently associated with T2DM in several ethnicities.^[13–16]^ Further, rs1799859 and rs757110 located in *ABCC8* were also selected, for two main reasons. First, these SNPs have been reported to be associated with T2DM and are predicted to affect sulfonylurea treatment.^[14,16–18]^ Second, the close location of these 4 SNPs on chromosome arm 11p15 provides useful information for linkage disequilibrium analysis. Therefore, this study sought to elucidate the potential associations of 4 SNPs (rs5219, rs2285676, rs1799859, and rs757110) with T2DM in a Kinh Vietnamese population.

## 2. Material and methods

### 2.1. Subject recruitment

We recruited 404 unrelated subjects who self-identified as Kinh Vietnamese at the University Medical Center. The study protocol was approved by the Ethical Committee of the University of Medicine and Pharmacy at Ho Chi Minh City (HEC/IRB number 350/HĐĐĐ-ĐHYD). All study subjects gave informed written consent before participating in the study. Subjects were considered to have T2DM if they had a history of T2DM or were newly diagnosed with T2DM according to the American Diabetes Association 2020 criteria.^[19]^

Exclusion criteria for T2DM subjects were: type 1 diabetes, liver dysfunction, use of drugs affecting plasma glucose levels, and endocrine diseases affecting plasma glucose levels. Non-diabetic control subjects were recruited from regular health checkup visitors at the University Medical Center. Exclusion criteria for control subjects were: previous history of diabetes of any type, use of substances affecting plasma glucose levels, pregnancy, cancer, and any other diseases affecting blood glucose levels. Finally, 202 T2DM and 202 control subjects were recruited.

### 2.2. Clinical and laboratory measurements

On recruitment, subjects went through a comprehensive physical examination, in addition to answering a survey on medical history relevant to the study. Relevant anthropometric measurements (including weight, height, waist circumference, hip circumference, and systolic and diastolic blood pressure) were obtained, along with demographic information (including age, sex, and duration of T2DM since diagnosis for T2DM subjects).

Subjects’ blood samples were taken if they met the criteria for having fasted for a minimum of 8 hours. Blood samples were immediately taken for biochemical analysis using a Beckman Coulter AU2700 Chemistry Analyzer. Laboratory measurements included plasma glucose, HbA1c, total serum cholesterol, high-density lipoprotein cholesterol, low-density lipoprotein cholesterol, triglycerides, and creatinine levels.

### 2.3. Genotyping

The remaining blood samples were stored at −20°C until extracted for genomic DNA using a QiAmp DNA Blood Mini Kit (QIAGEN, Hilden, Germany). The genotyping of rs5219 was performed as previously described.^[20]^ Rs2285676 was genotyped using restriction fragment length polymorphism polymerase chain reaction (PCR) with BcnI enzyme (Thermo Scientific, Waltham, MA, United States). The detailed protocol for genotyping rs2285676 is described in Supplementary Table S1, http://links.lww.com/MD/H879. Rs757110 and rs1799859 were genotyped using a tetra-primer amplification refractory mutation system PCR. The sequence of primers and their ratios in the PCR mix are listed in Supplementary Table S2, http://links.lww.com/MD/H880. All the PCRs were performed with Takara Taq polymerase (TakaraBio, San Jose, CA, United States) in a SimpliAmp thermal cycler (Thermo Scientific) under the following conditions: initial denaturation at 98°C for 3 minutes, followed by 30 cycles of 98°C for 15 seconds (denaturation), 60°C for 20 seconds (annealing), 72°C for 40 seconds (elongation), and 72°C for 2 minutes (final elongation).

Thirty random DNA samples were chosen for direct sequencing of the genetic regions containing the 4 SNPs. The protocol for direct sequencing was described previously.^[21–23]^ The results of sequencing were used as controls for the compatibility of PCR genotyping.

### 2.4. Statistical analyses

The clinical characteristics of the T2DM and control groups were statistically analyzed using Student’s independent 2-tailed *t* test and the Chi-Square test for independence. The ANOVA one-way test was used to compare differences in means between >2 groups.

Genotype frequencies were assessed for being under Hardy-Weinberg equilibrium (HWE) using the goodness-of-fit Chi-Square test. The web tool SNPstats was used to test for potential associations of *KCNJ11* SNPs rs5219, rs2285676, rs1799859, and rs757110 with T2DM.^[24]^ Multiple inheritance models were used in the association tests: codominant, dominant, recessive and log-additive. To mitigate covariate effects in the analysis, age, sex, and BMI were used as covariates for statistical adjustment. Odds ratios were calculated as 95% confidence intervals (CI). Haplotype frequencies and linkage disequilibrium for allele pairs were analyzed using the Python SciKit-Allel package.^[25]^ In this study, 2-sided *P* values < .05 were considered statistically significant.

## 3. Results

### 3.1. Clinical and biochemical characteristics of studied subjects

Baseline demographic and clinical measurements for T2DM and control subjects are presented in Table 1. Statistically significant differences between the T2DM and control populations were found in the following measurements: HbA1c, FPG, diastolic blood pressure, total cholesterol, high-density lipoprotein cholesterol, and low-density lipoprotein cholesterol (*P* < .05). Meanwhile, there were no statistically significant differences between the 2 populations for the following measurements: sex, age, BMI, waist circumference, waist-to-hip ratio, systolic blood pressure, triglyceride levels, and serum creatinine levels (*P* > .05).

**Table 1:** Baseline clinical and biochemical characteristics of the studied population.

	T2DM N = 202	Controls N = 202	*P* value
Males/females	66/136	73/129	.53
Duration of disease (yr)	5.17 ± 5.16	–	n/a
Age at diagnosis (T2DM)/age at recruitment (controls)	54.92 ± 6.34	55.28 ± 5.97	.56
BMI (kg/m^2^)	24.23 ± 3.07	23.23 ± 3.13	.99
Waist circumference (cm)	84.76 ± 9.43	83.97 ± 8.94	.39
WHR	0.91 ± 0.07	0.91 ± 0.07	.44
HbA1c (%)	8.25 ± 2.18	5.68 ± 0.43	<.001*
FPG (mmol/L)	8.22 ± 2.62	5.63 ± 0.52	<.001*
SBP (mm Hg)	130.46 ± 18.04	130.20 ± 16.02	.88
DBP (mm Hg)	78.07 ± 10.92	81.45 ± 10.71	<.01*
Triglycerides (mmol/L)	2.39 ± 1.66	2.13 ± 1.50	.10
Total cholesterol (mmol/L)	4.67 ± 1.39	5.50 ± 1.23	<.001*
HDL cholesterol (mmol/L)	1.15 ± 0.31	1.29 ± 0.46	<.001*
LDL cholesterol (mmol/L)	2.99 ± 1.00	3.57 ± 0.95	<.001*
Serum creatinine (mg/dL)	0.87 ± 0.21	0.84 ± 0.20	.25

BMI = body mass index, DBP = diastolic blood pressure, FPG = fasting plasma glucose, HDL = high-density lipoprotein, LDL = low-density lipoprotein, SBP = systolic blood pressure, T2DM = type 2 diabetes mellitus, WHR = waist-hip ratio.

* Statistically significant.

### 3.2. Association of KCNJ11 and ABCC8 SNPs with T2DM

Overall, genotype frequencies for rs5219, rs2285676, rs1799859, and rs757110 were under HWE in the studied population, though T2DM in rs1799859 was not, according to the exact test for HWE (Supplementary Table S3, http://links.lww.com/MD/H881). Multiple inheritance models were used to assess statistically significant associations between genotypes and T2DM status. Odds ratios were calculated with 95% CIs. Calculations were statistically adjusted using age, sex, and BMI to mitigate covariate effects.

Of the 4 SNPs studied, rs5219 showed a statistically significant association for its A allele in the codominant and recessive models (Table 2). Of note, within the codominant model of inheritance, rs5219 showed a statistically significant association only within the A/A allele; that is, overall, when calculating the inheritance model with the A/G allele, it is not statistically significant (*P* > .05). The other 3 SNPs (rs2285676, rs1799859, and rs757110) did not show any statistically significant association with T2DM phenotype (Supplementary Table S4, http://links.lww.com/MD/H882). As AA genotype of rs1799859 was not identified in this population, the co-dominant and recessive models were not analyzed.

**Table 2:** The association of rs5219 with T2DM.

Model	Genotype	Control (n)	T2DM (n)	Adjusted OR (95% CI)†	*P* value†
Co-dominant	G/G	92	82	1	.07
	A/G	94	89	1.07 (0.71–1.62)	
	A/A	16	31	2.15 (1.09–4.22)*	
Dominant	G/G	92	82	1	.31
	A/G, A/A	110	120	1.23 (0.82–1.82)	
Recessive	G/G, A/G	186	171	1	.02*
	A/A	16	31	2.08 (1.09–3.94)*	
Log-additive	–	–	–	1.32 (0.98–1.77)	.07

n = 404.

OR = odds ratio, CI = confidence interval, T2DM = type 2 diabetes mellitus.

* Statistically significant.

† Adjusted for sex, age, and body mass index (BMI).

### 3.3. Haplotype analysis

**Figure 1.**
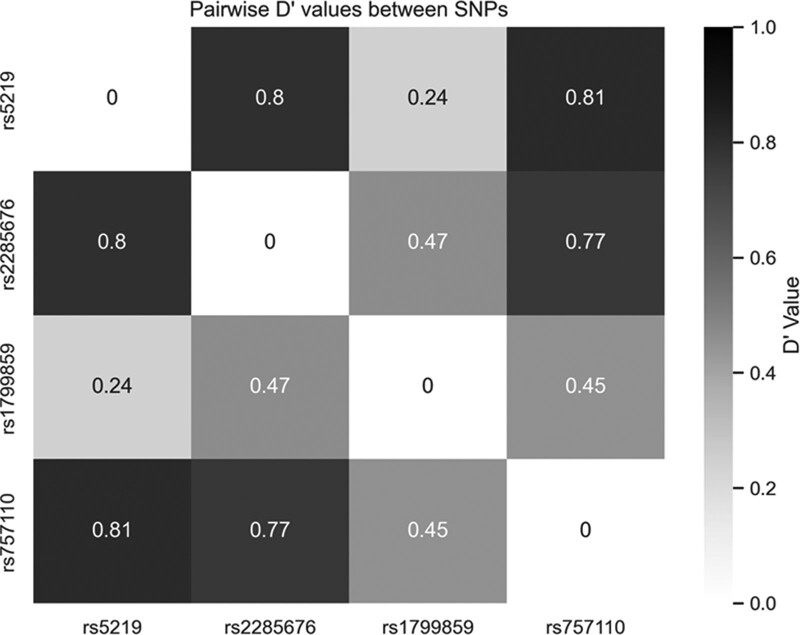
Linkage disequilibrium between allele pairs of rs5219, rs2285676, rs1799859, and rs757110.

Strong linkage disequilibrium was observed between rs5219, rs2285676, and rs757110 (Fig. 1). With this observation, we attempted to find potential associations of T2DM to haplotypes for rs5219, rs2285676, and rs757110 (Table 3). In line with our observations of the A allele for rs5219, we observed a statistically significant association of T2DM with the rs5219(A)/rs2285676(T)/rs757110(G) haplotype.

**Table 3:** The association of haplotype rs5219/rs2285676/rs757110 with T2DM.

rs5219	rs2285676	rs757110	Frequency	Adjusted OR (95% CI)†	*P* value†
G	C	T	0.5142	1.00	–
A	T	G	0.2844	1.42 (1.01–1.99)*	.04*
G	T	T	0.0875	0.90 (0.54–1.50)	.68
G	C	G	0.0288	1.42 (0.60–3.33)	.42
G	T	G	0.0267	1.36 (0.59–3.12)	.47
A	C	T	0.0221	2.16 (0.77–6.00)	.14
A	T	T	0.0185	0.87 (0.28–2.67)	.80
A	C	G	0.0178	0.51 (0.15–1.72)	.28

N = 404.

OR = odds ratio, CI = confidence interval, T2DM = type 2 diabetes mellitus.

* Statistically significant.

† Adjusted for sex, age, and BMI.

### 3.4. Association of rs5219 to clinical and biochemical characteristics

Because the A allele of rs5219 was found to be associated with T2DM, we further analyzed subjects’ clinical and biochemical characteristics with respect to genotype (Table 4). We observed a statistically significant difference between subjects’ genotypes for diastolic blood pressure. However, overall, no particularly meaningful differences were observed in the subjects with respect to their genotypes in the context of T2DM.

**Table 4:** The association of rs5219 with clinical and biochemical characteristics in T2DM and control subjects.

Characteristics	rs5219											
	T2DM (N = 202)				Controls (N = 202)				Studied population (N = 404)			
	GG	GA	AA	ANOVA *P* value	GG	GA	AA	ANOVA *P* value	GG	GA	AA	ANOVA *P* value
BMI (kg/m^2^)	24.24 ± 3.27	24.24 ± 2.59	24.17 ± 3.70	.99	24.22 ± 2.96	24.20 ± 3.42	24.45 ± 2.12	.96	24.23 ± 3.11	24.22 ± 3.05	24.26 ± 3.25	1.00
WHR	0.91 ± 0.06	0.91 ± 0.06	0.92 ± 0.11	.86	0.92 ± 0.08	0.90 ± 0.06	0.91 ± 0.06	.43	0.91 ± 0.07	0.91 ± 0.06	0.92 ± 0.09	.72
HbA1c (%)	8.09 ± 2.05	8.38 ± 2.27	8.27 ± 2.23	.92	5.64 ± 0.46	5.74 ± 0.46	5.83 ± 0.39	.78	8.01 ± 2.11	8.08 ± 2.22	8.17 ± 1.89	.89
FPG (mmol/L)	7.89 ± 2.24	8.45 ± 2.81	8.38 ± 2.90	.36	5.63 ± 0.52	5.66 ± 0.53	5.50 ± 0.48	.51	6.69 ± 1.95	7.02 ± 2.43	7.40 ± 2.73	.13
SBP (mm Hg)	130.63 ± 16.71	130.34 ± 20.39	130.35 ± 13.70	.99	133.21 ± 15.43	126.56 ± 15.85	134.38 ± 15.78	.10	131.98 ± 16.10	128.41 ± 18.31	131.72 ± 14.57	.12
DBP (mm Hg)	78.88 ± 9.72	77.60 ± 12.48	77.29 ± 8.88	.68	78.38 ± 9.78	84.58 ± 10.91	81.75 ± 9.56	<.001*	81.86 ± 10.75	77.99 ± 11.19	78.81 ± 9.30	<.01*
Triglycerides (mmol/L)	2.42 ± 1.69	2.34 ± 1.65	2.47 ± 1.59	.92	2.40 ± 1.93	1.90 ± 1.01	2.00 ± 0.68	.08	2.41 ± 1.82	2.12 ± 1.38	2.31 ± 1.37	.22
Total cholesterol (mmol/L)	4.52 ± 1.40	4.74 ± 1.41	4.88 ± 1.33	1.42	5.60 ± 1.28	5.40 ± 1.16	5.56 ± 1.25	.53	5.09 ± 1.44	5.08 ± 1.33	5.12 ± 1.34	.98
HDL cholesterol (mmol/L)	1.11 ± 0.26	1.20 ± 0.35	1.11 ± 0.23	.13	1.26 ± 0.29	1.32 ± 0.60	1.33 ± 0.27	.63	1.19 ± 0.29	1.26 ± 0.50	1.19 ± 0.27	.19
LDL cholesterol (mmol/L)	2.79 ± 0.97	3.01 ± 1.03	2.97 ± 0.95	.36	3.57 ± 0.98	3.54 ± 0.94	3.71 ± 0.88	.82	3.20 ± 1.05	3.28 ± 1.02	3.22 ± 0.99	.77

BMI = body mass index, DBP = diastolic blood pressure, HDL = high-density lipoprotein, LDL = low-density lipoprotein, PG = fasting plasma glucose, SBP = systolic blood pressure, T2DM = type 2 diabetes mellitus, WHR = waist-hip ratio.

* Statistically significant.

## 4. Discussion

T2DM is an increasingly burdensome global epidemic, highlighting the need to investigate genetic risk loci for T2DM. With the adoption of precision medicines for diseases such as T2DM,^[26]^ effort must be devoted to building the genomic datasets that will inform the use of these medicines. We sought to contribute to the literature on the T2DM genetic landscape in Kinh Vietnamese with this study, investigating SNPs located in *KCNJ11* and *ABCC8*.

The study’s results suggest that the A allele of rs5219 is associated with T2DM in Kinh Vietnamese. This finding is in line with observations in other populations, such as within the Caucasian DESIR cohort and an ethnic Han Chinese population.^[13,15,27]^ However, this association is not consistent in studies performed on Japanese populations.^[14,28]^

The other SNPs studied—rs2285676, rs1799859, and rs757110—were not found to be associated with T2DM in the Kinh Vietnamese population studied, although these SNPs have been shown to be associated with T2DM in populations such as South Indian, Chinese, and Japanese.^[14,16,18]^ In a study carried out with a cohort of Kurdish individuals from western Iran, the G allele of rs757110 was found to be associated with T2DM, increased homeostatic model assessment of insulin resistance, and hyperinsulinemia.^[29]^ The study’s authors consider that the association is due to the effects of the functional mutation from alanine (position 1369) to serine. However, within our studied population, we observed no such effects. Another interesting finding of this study is that the strong linkage disequilibrium between pairs of SNPs rs5219, rs2285676, rs757110 and the rs5219(A)/rs2285676(T)/rs757110(G) haplotype is statistically associated with T2DM. Given that these linkages were not investigated in the previously mentioned studies of rs757110^[18,29]^ and that rs5219 is a leading SNP that is consistently associated with T2DM, the association of rs757110 with T2DM in certain populations may be partially explained by this strong linkage equilibrium.

The discrepancies in these findings once again highlight the importance of accounting for interethnic differences in genetic studies. For example, it has been suggested that the Kinh Vietnamese population carries potentially unique genotype-phenotype associations.^[30–33]^ For a country with approximately 100 million inhabitants and increasingly facing the challenges of T2DM,^[34]^ genetic associations with T2DM in Kinh Vietnamese is important information that is worth studying.

This study has some limitations. First, no functional characterization was performed on subjects presenting with the rs5219(G) alleles or rs5219(A)/rs2285676(T)/rs757110(G) haplotype, especially the measurement of the HOMA-B index. Our associations are thus strictly within the context of genotype. Second, the study’s sample size was relatively small. Nevertheless, future meta-analyses and functional studies building on this study may help strengthen its findings and may also reconcile the observed interethnic differences in *KCNJ11* and *ABCC8* genotype-phenotype associations in the literature.

In conclusion, we have found that rs5219 is a strong lead candidate SNP associated with T2DM in the Kinh Vietnamese, while no associations were found for rs2285676, rs1799859, and rs757110. Further genetic association studies between *KCNJ11, ABCC8* SNPs and T2DM are required in a larger Kinh Vietnamese population to investigate further the genetic contribution of insulin deficiency mechanisms in T2DM.

## Author contributions

**Conceptualization:** Thao Phuong Mai, Minh Duc Do.

**Data curation:** Nam Quang Tran, Phat Tung Ma, Chi Khanh Hoang, Bao Hoang Le, Thang Tat Ngo Dinh, Luong Van Tran, Thang Viet Tran.

**Formal analysis:** Steven D. Truong, Minh Duc Do.

**Funding acquisition:** Minh Duc Do.

**Investigation:** Linh Hoang Gia Le, Khuong Thai Le, Hien Thanh Nguyen, Hoang Anh Vu.

**Methodology:** Nam Quang Tran, Minh Duc Do.

**Project administration:** Minh Duc Do.

**Resources:** Minh Duc Do.

**Software:** Steven D. Truong, Minh Duc Do.

**Supervision:** Minh Duc Do.

**Validation:** Hoang Anh Vu, Minh Duc Do.

**Visualization:** Steven D. Truong.

**Writing – original draft:** Steven D. Truong, Minh Duc Do.

**Writing – review & editing:** Thao Phuong Mai, Minh Duc Do.

## Supplementary Material
