# Supplementary material for: Association of KCNJ11 and ABCC8 single-nucleotide polymorphisms with type 2 diabetes mellitus in a Kinh Vietnamese population
Source: Medicine (Baltimore). 2022 Nov 18;101(46):e31653. doi: 10.1097/MD.0000000000031653 (PMC9678638; doi:10.1097/MD.0000000000031653)
Supplement: Supplementary file 1 [file medi-101-e31653-s001.pdf]

Supplementary Table 1. Protocol for rs2285676 genotyping

Primer sequence (5'-3')	Fragment length after PCR and digestion by BcnI (bp)		
F: 5’-CAGGAACCTGTACTGGGTTA-3’	C/C	T/T	T/C
R: 5’-AATCATGTGTGGTGGGCACA-3’	356 + 276	632	632 + 356 + 276
