# Supplementary material for: Association of KCNJ11 and ABCC8 single-nucleotide polymorphisms with type 2 diabetes mellitus in a Kinh Vietnamese population
Source: Medicine (Baltimore). 2022 Nov 18;101(46):e31653. doi: 10.1097/MD.0000000000031653 (PMC9678638; doi:10.1097/MD.0000000000031653)
Supplement: Supplementary file 2 [file medi-101-e31653-s002.pdf]

Supplementary Table 2. Primer sequence and their ratio for rs757110 and rs1799859 genotyping

SNP	Primer sequence	Primer ratio	Fragment length after PCR (bp)		
rs757110	F1: 5'-AACTGGATGGTGAGGAACCT-3'	F1:T-R:G-F:R (1:3:1:0.5)	G/G	T/T	G/T
	T-R: 5'-CTGACCTTCTGTCCAGGAGA-3'				
	G-F: 5'-GCACGTCAATGCCCTCATCG-3'		541 + 173	541 + 368	541 + 368 + 173
	R: 5'-AGGAGACTGCGATGTCTGAA-3'				
rs1799859	F1: 5'-GTGACGTGTGCATGAGTTGC-3'	F1:G-R1:A-F2:R (1:0.25:6:3)	G/G	A/A	G/A
	G-R1: 5'-CCAGGCCAGCAGAGAGCTCC-3'				
	A-F2: 5'-TCTCCAACTCCCTGCACTG-3'		593 + 220	593 + 373	593 + 373 + 220
	R: 5' -GCTCTTCTTAAGGCTGGAGA-3'				
