# Supplementary material for: Association of KCNJ11 and ABCC8 single-nucleotide polymorphisms with type 2 diabetes mellitus in a Kinh Vietnamese population
Source: Medicine (Baltimore). 2022 Nov 18;101(46):e31653. doi: 10.1097/MD.0000000000031653 (PMC9678638; doi:10.1097/MD.0000000000031653)
Supplement: Supplementary file 3 [file medi-101-e31653-s003.pdf]

Supplementary Table 3: Genotype frequencies and Hardy-Weinberg equilibrium.

rs5219						
	n11	n12	n2	n1	n2	*P*-value
Total	172	183	47	531	277	1.00
Control	92	94	16	278	126	0.26
T2DM	82	89	31	253	151	0.45
rs2285676						
Total	140	191	73	471	337	0.61
Control	73	99	30	245	159	0.77
T2DM	67	92	43	226	178	0.32
rs1799859						
Total	347	52	5	746	62	0.07
Control	179	23	0	381	23	1.00
T2DM	168	29	5	365	39	0.02*
rs757110						
Total	165	189	50	519	289	0.75
Control	89	94	19	272	132	0.52
T2DM	76	95	31	247	157	0.88

T2DM: type 2 diabetes mellitus. N = 404.

*Statistically significant
