# Supplementary material for: Association of KCNJ11 and ABCC8 single-nucleotide polymorphisms with type 2 diabetes mellitus in a Kinh Vietnamese population
Source: Medicine (Baltimore). 2022 Nov 18;101(46):e31653. doi: 10.1097/MD.0000000000031653 (PMC9678638; doi:10.1097/MD.0000000000031653)
Supplement: Supplementary file 4 [file medi-101-e31653-s004.pdf]

Supplementary Table 4. Association of rs2285676, rs1799859, and rs757110 with T2DM

Rs2285676	Genotype	Control (n)	T2DM (n)	Adjusted OR (95% CI)†	*P*-value†
Co-dominant	C/C	73	67	1	0.26
	C/T	99	92	1.02 (0.66-1.58)	
	T/T	30	43	1.55 (0.87-2.76)	
Dominant	C/C	73	67	1	0.53
	C/T, T/T	129	135	1.14 (0.76-1.72)	
Recessive	C/C, C/T	172	159	1	0.10
	T/T	30	43	1.54 (0.92-2.57)	
Log-additive	—	—	—	1.21 (0.91-1.59)	0.19
Rs1799859	Genotype	Control (n)	T2DM (n)	Adjusted OR (95% CI)†	*P*-value†
Co-dominant	G/G	179	168	1	NA
	G/A	23	29	1.33 (0.74-2.40)	
	A/A	0	5	NA (0.00-NA)	
Dominant	G/G	179	168	1	0.12
	G/A, A/A	23	34	1.56 (0.88-2.77)	
Recessive	G/G, G/A	202	197	1	NA
	A/A	0	5	NA (0.00-NA)	
Log-additive	—	—	—	1.69 (1.00-2.84)	0.05
Rs757110	Genotype	Control (n)	T2DM (n)	Adjusted OR (95% CI)†	*P*-value†
Co-dominant	T/T	89	76	1	0.14
	T/G	94	95	1.19 (0.78-1.81)	
	G/G	19	31	1.92 (1.00-3.67)	
Dominant	T/T	89	76	1	0.18

	T/G, G/G	113	126	1.31 (0.88-1.96)	
Recessive	T/T, T/G	183	171	1	0.07
	G/G	19	31	1.75 (0.95-3.22)	
Log-additive	—	—	—	1.32 (0.98-1.77)	0.06

† Adjusted for sex, age, and BMI.

OR: odds ratio, CI: confidence interval, T2DM: type 2 diabetes mellitus. N = 404.

*Statistically significant
